# A unique intraluminal growth of juvenile nasopharyngeal angiofibroma: A case report

**DOI:** 10.37796/2211-8039.1019

**Published:** 2020-09-01

**Authors:** Mojtaba Mohammadi Ardehali, Shirin Irani, Mohammadreza Firouzifar

**Affiliations:** Otorhinolaryngology Research Center, Amir Alam Hospital, Tehran University of Medical Sciences, Tehran, Iran

**Keywords:** Angiofibroma, sinus endoscopy, intraluminal growth

## Abstract

Juvenile nasopharyngeal angiofibroma (JNA) is a rare, hypervascular, benign tumor which is mainly seen among male adolescents. The tumor typically originates from the sphenopalatine fossa, but could spread through natural foramens and fissures.

There are some reports of atypical growth of this tumor in literature but the intraluminal growth, which could be seen in paraganglioma and glomus tumors, has not reported yet in angiofibroma. In this article we present a case of extensive angiofoibroma with intraluminal involvement of the ophthalmic vein.

Our patient was a 19-year-old boy with a complaint of nasal obstruction and occasional epistaxis since a year ago, without any visual or neurologic complaints. The patient underwent an endoscopic resection of the tumor after embolization via the nasal cavity.

The intraoperative findings revealed the tumor extension to the orbit, intracranial space and cavernous sinus via inferior orbital fissure. The intracranial extension of the tumor was extradural and was successfully excised without CSF leakage. An interesting finding in this patient, was an intraluminal extension of the tumor in to the ophthalmic vein, which was completely excised endoscopically. (pre and post operation pictures are available in the full text). The definitive treatment of angiofibroma is surgical excision. Different surgical approaches are used but nowadays endoscopic resection with or without pre-operative embolization is the first choice of treatment. The intraluminal growth of the tumor was also excised as a pedunculated mass separately.

## Introduction

Juvenile nasopharyngeal angiofibroma (JNA) is a rare, hypervascular, benign tumor which is mainly seen among male adolescents [1]. It accounts for 0.05% of head and neck tumors. Despite its benign histologic pattern, it could have an aggressive clinical trend to invade adjacent anatomic structures [2].

The tumor typically originates from the sphenopalatine fossa, but could spread through natural foramens and fissures. It could involve nasopharynx, the nasal cavity, maxillary and ethmoid sinuses by medial extension. Lateral growth results in pterygopalatine fossa involvement and furtherly extending to infratemporal fossa and cheek via pterygomaxillary fissure.

It could involve the orbit via the inferior orbital fissure from the infratemporal fossa. Also, tumor growth to the parasellar compartment of the middle cranial fossa is seen by means of superior orbital fissure.

Additionally, the encasement of the internal carotid artery and invasion of the cavernous sinus may be seen in advanced intracranial involvement [2].

There are some reports of atypical growth of this tumor in literature but the intraluminal growth, which could be seen in paraganglioma and glomus tumors [3], has not reported yet in angiofibroma. In this article we present a case of extensive angiofoibroma with intraluminal involvement of the ophthalmic vein.

## Case presentation

A 19-year-old boy referred to our clinic by a complaint of nasal obstruction since a year ago. He complained of progressive obstruction of the right nasal side which extends to bilateral obstruction gradually. He also complained of occasional epistaxis. He did not have any visual or neurologic complaints. The past medical history, family history and drug history was unremarkable.

Rhinoscopic examination, revealed a mass in right nasal cavity which occlude the nose. He had also a mild swelling of the cheek and mild proptosis of the right eye. The visual acuity was detected 10/10 in ophthalmology consultation. Marcus gunn sign was negative and the eye movements were all normal.

The patient has also undergone a pre-operation neurosurgical consultation. The neurological evaluation was unremarkable with normal cranial nerves' function.

**Fig. 1:**
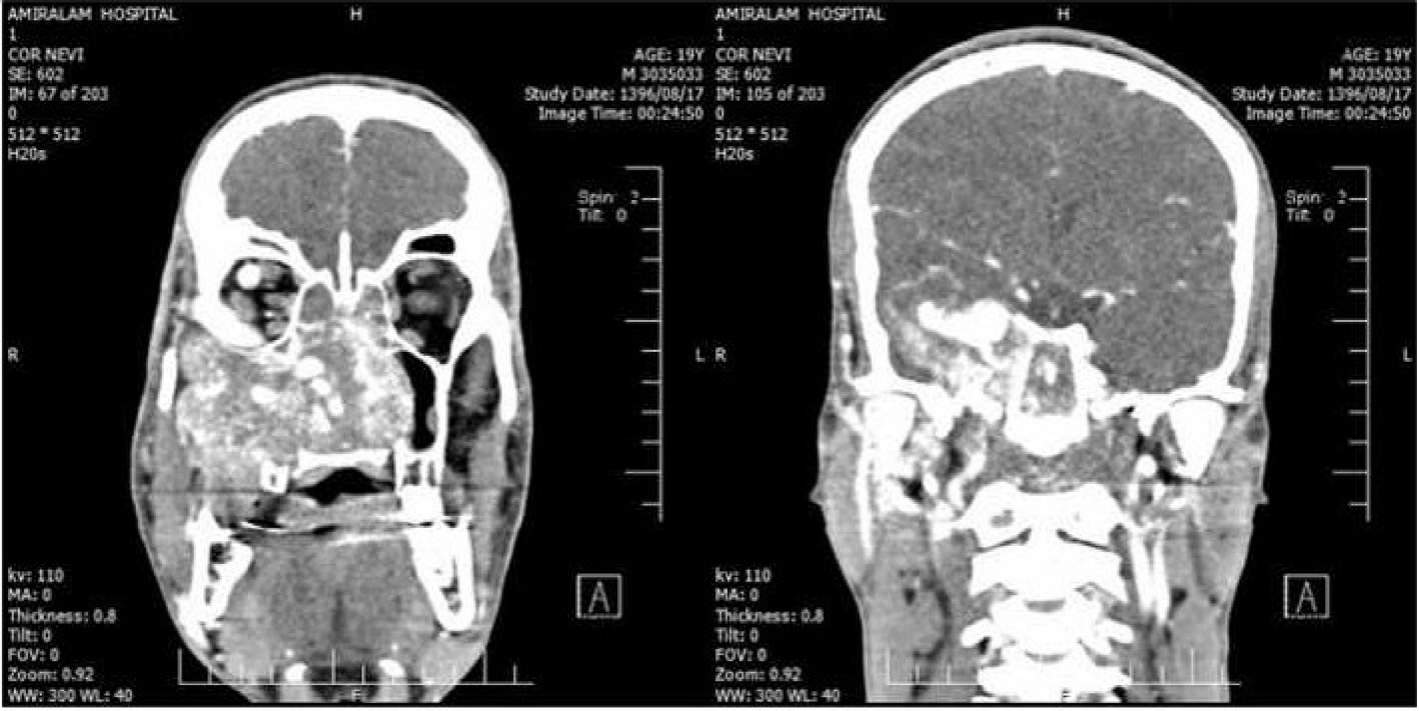
The computed tomography scan with intravenous contrast revealed a large mass in right nasal cavity with destruction of pterygoid plates, the body and the greater wing of the sphenoid, inferior orbital wall and the posterior wall of the maxillary sinus.

**Fig. 2:**
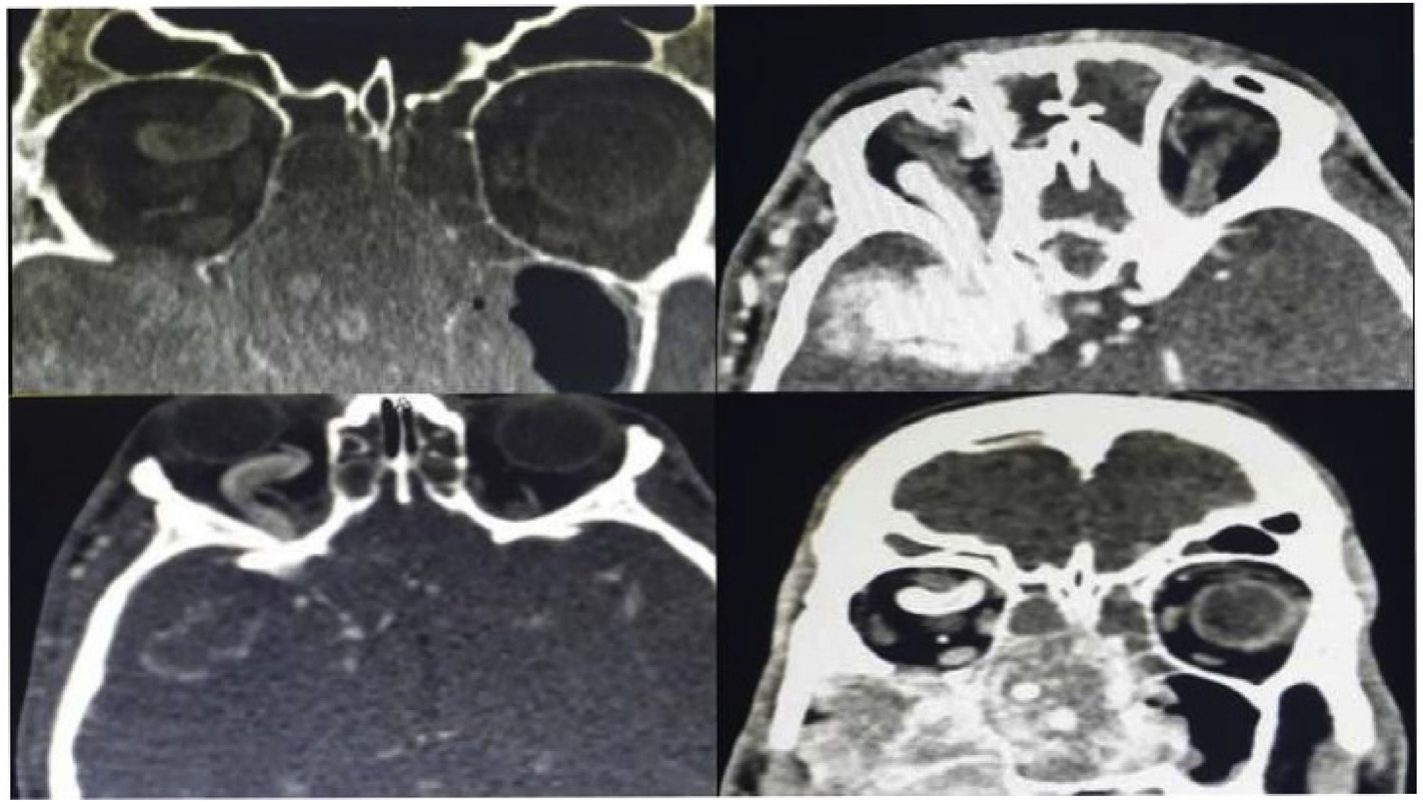
The computed tomography scan with intravenous contrast revealed a large hypervascular mass in right nasal cavity with orbital involvement.

The computed tomography scan (CT scan) revealed a large mass in right nasal cavity with destruction of pterygoid plates, the body and the greater wing of the sphenoid, inferior orbital wall and the posterior wall of the maxillary sinus (Fig. 1). There was also evidences of intracranial extension, infratemporal invasion and extension to nasopharynx in the imaging. The mass was so close to right orbital apex. Large vascular structures were noticed in the scan. Also, there was a dilatation of superior ophthalmic vein, facial vein and ethmoidal veins (Fig. 2).

**Fig. 3:**
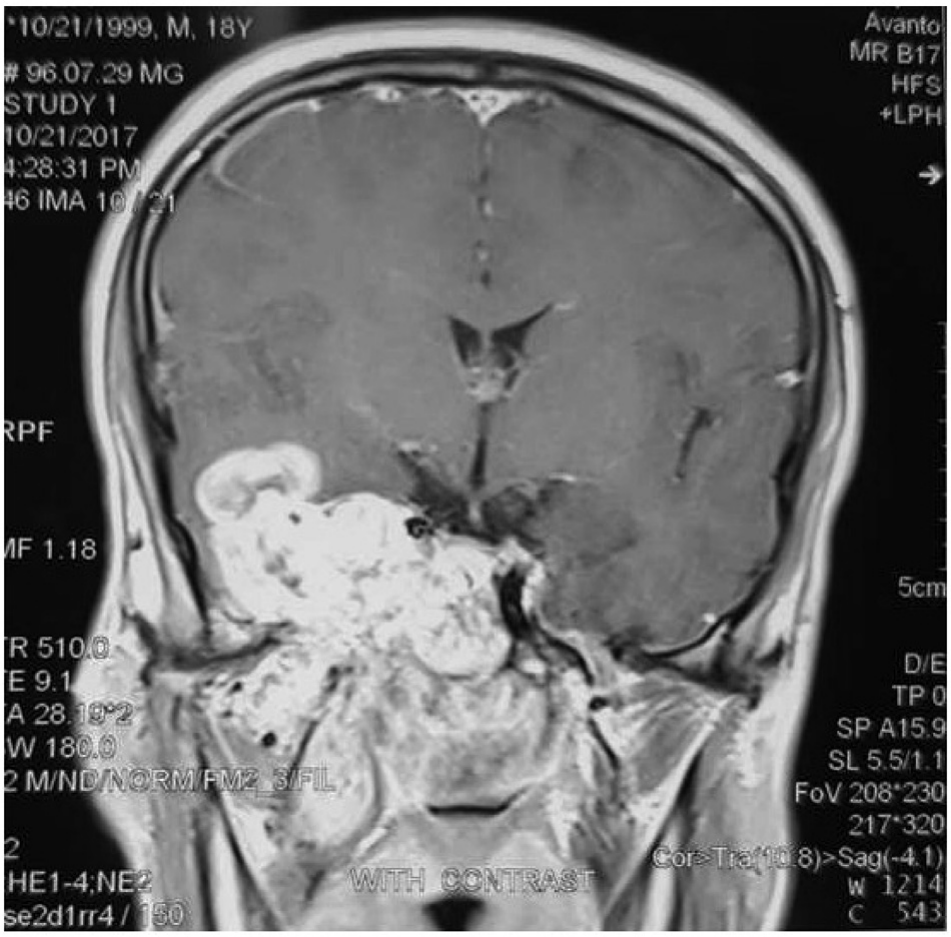
MRI showed a hypervascular mass with plenty of flow voids signal, due to containing large vessels, in the right nasal cavity with extension to intracranial and infratemporal spaces.

The Magnetic Resonance Imaging (MRI), also endorsed the evidences of CT scan (Fig. 3). A hypervascular mass with plenty of flow voids signal, due to containing large vessels, was detected in the right nasal cavity with extension to intracranial and infratemporal spaces.

**Fig. 4:**
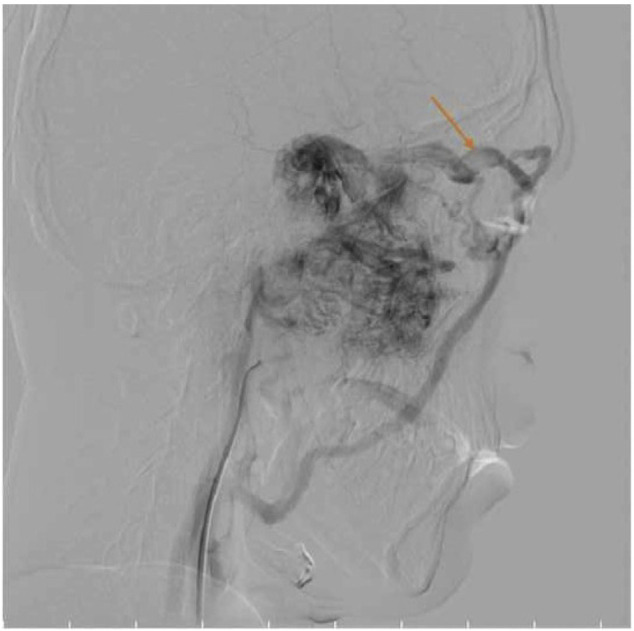
The angiographic study showed a filling defect in ophthalmic vein according to intraluminal tumor growth.

The patient was scheduled for endoscopic resection of the tumor, by the first impression of angiofibroma, Radkowski stage of IIIB. Due to its vast extension, the pre-operation embolization was planned 48 hours before surgery. The feeding vessels of the JNA was bilateral internal maxillary arteries and bilateral internal carotid arteries. The bilateral internal maxillary arteries were embolized by PVA 300-350. The intralumininal growth is depicted in the angiographic examination (Fig. 4).

The patient underwent an endoscopic resection of the tumor via the nasal cavity. For optimal access to lateral border of the tumor, a gingivobuccal incision was performed.

**Fig. 5:**
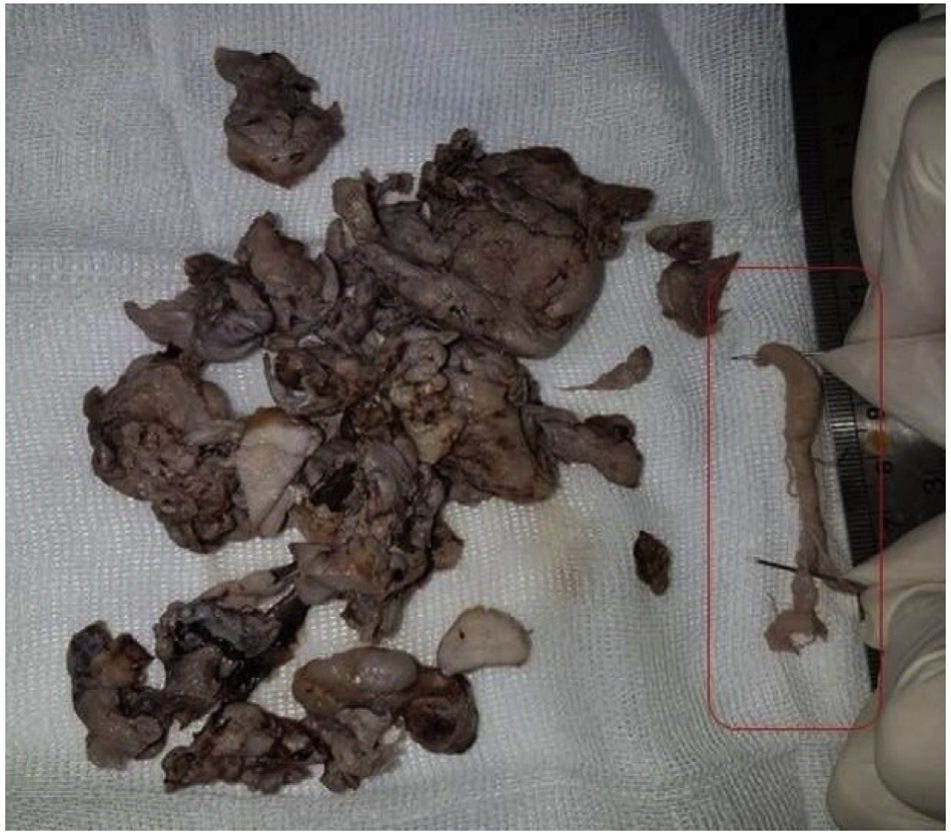
The excised specimen with an intraluminal extension in to ophthalmic vein (marked in the red box), which was completely excised endoscopically.

The intraoperative findings revealed that the tumor extends to orbit, intracranial space and cavernous sinus via inferior orbital fissure. The intracranial extension of the tumor was extradural and was successfully excised without CSF leakage. An interesting finding in this patient, was an intraluminal extension of the tumor in to ophthalmic vein, which was identified intra-operatively and completely excised endoscopically (Fig. 5). The blood loss volume was 1400 cc and he received 4 pack cell during the endoscopic procedure. The hemoglobin level was 12.7 g/dl pre-operatively, while it was 11.7 g/dl six hours after blood trans-fusion. The neurologic examination and visual acuity was intact post operatively. The post-operative period was unremarkable and he was discharged 4 days later. The patient had a recurrence tumor a year later in the lateral portion of the maxilla which was excised endoscopically and did not need other ancillary treatments. The pathology evaluation, confirmed the diagnosis of angiofibroma.

## Discussion

Juvenile nasopharyngeal angiofibroma is a histologically benign tumor but could have an aggressive behaviour and can invade contiguous structures [4].

The usual pattern of JNA growth is known, but there are some reports about unusual or atypical types of angiofibroma. Morkenborg and his colleagues have presented a case of bilateral angiofibroma which were completely removed by embolization followed by endoscopic surgery [5]. Pasalic has introduced a rare case of supratentorial and infratentorial localized angiofibroma who was initially presented with symptoms of increased intracranial pressure [6].

Celik et al presented four atypical cases of angiofibroma with unusual localization of the tumor like tonsillar bed or inferior concha [4]. Vasconcelos AC et al reported an atypical angiofibroma with a soft palate lesion [7].

But we did not find any report about intraluminal growth of this tumor. This phenomenon is occasionally seen in glomus jugular and there are some case reports about it [3]. But, it has not been reported in literature.

The definitive treatment of angiofibroma is surgical excision. Different surgical approaches are used but nowadays endoscopic resection with or without pre-operative embolization is the first choice of treatment. Although the tumors that involve multiple compartments can be challenging to access surgically [8].

We performed an embolization followed by endoscopic resection due to extensive tumor and it was successfully excised via endoscopic surgery. The intraluminal growth of the tumor was also excised as a pedunculated mass separately (Fig. 4).

## Conclusion

Juvenile angiofibroma which is a rare benign tumor with aggressive growth pattern, can invade adjacent critical structures like intracranial or infratemporal fossa. In this manuscript, we present a unique intraluminal growth of the tumor into the ophthalmic vein, which should be keep in mind as one way of its extension.
